# The prevalence of malaria at first antenatal visit in Blantyre, Malawi declined following a universal bed net campaign

**DOI:** 10.1186/s12936-015-0945-3

**Published:** 2015-10-29

**Authors:** Sarah Boudová, Titus Divala, Patricia Mawindo, Lauren Cohee, Linda Kalilani-Phiri, Phillip Thesing, Terrie E. Taylor, Miriam K. Laufer

**Affiliations:** Division of Malaria Research, Institute for Global Health, University of Maryland School of Medicine, Baltimore, MD USA; Blantyre Malaria Project, University of Malawi College of Medicine, Blantyre, Malawi; University of Malawi College of Medicine, Blantyre, Malawi; Department of Osteopathic Medical Specialties, College of Osteopathic Medicine, Michigan State University, East Lansing, MI USA

**Keywords:** Malaria, Pregnancy, Malawi, Bed net

## Abstract

**Background:**

Preventing malaria during pregnancy is important for the health of mothers and newborns. Interventions, which include distribution of bed nets and administration of intermittent preventive treatment (IPT), typically occur at the first antenatal visit, usually in the second or third trimester of pregnancy. In 2012, during the course of ongoing clinical studies of malaria among pregnant women in Malawi, a universal bed net campaign was implemented by the Government. This study tested the hypothesis that a universal bed net campaign would decrease the prevalence of malaria among pregnant women at their first antenatal visit.

**Methods:**

Some 1661 women were recruited for two studies from 2009 to 2014. Quantitative PCR (qPCR) was conducted from dried blood spots collected at the first antenatal care visit (prior to administration of IPT or any study interventions) from women who were in their first or second pregnancy and less than 28 weeks gestation by clinical assessment.

**Results:**

Overall, 320 of 1629 (19.6 %) women tested for malaria at their first antenatal visit were infected. Malaria infection rates declined from 28.4 % before the universal bed net campaign, to 18.5 % in 2012, to 15.0 % in the years following the universal bed net campaign. The odds of malaria infection at the time of first antenatal visit in 2012 and the years following the bed net campaign were significantly lower than in the years prior to the intervention (OR 0.6, 95 % CI 0.4–0.8; and OR 0.4, 95 % CI 0.3–0.6, respectively). A similar pattern was observed for the prevalence of clinical malaria. The inverse trend was observed for reported bed net use. However bed net use and malaria infection were not significantly associated on the individual level.

**Conclusions:**

Malaria infection in pregnant women is common even after a bed net campaign in Malawi, though prevalence rates declined. These early infections may cause maternal anaemia and placental malaria resulting in adverse maternal and fetal outcomes. Infection early in pregnancy may also contribute to malaria transmission as pregnant women represent a significant untreated reservoir of parasites. Universal bed net distribution appears to have moderate success in preventing malaria early in pregnancy and these findings support continued efforts to target women early in pregnancy and all women of childbearing age.

## Background

Every year 125 million pregnancies occur in malaria-endemic countries [1]. Pregnancy-associated malaria can result in maternal anaemia, low birth weight, prematurity, and increased infant mortality [2–8]. To prevent these health consequences, antenatal clinics distribute bed nets and provide intermittent preventive therapy (IPT) with sulfadoxine-pyrimethamine. However, women in sub-Saharan Africa typically seek out antenatal care late in pregnancy [9, 10], thus women are at risk of infection for months before receiving these interventions. Malaria infection during pregnancy is most prevalent at the time of the first antenatal visit [11–13] and these infections early in pregnancy have been associated with consequences for maternal and fetal well-being such as low birthweight, maternal anaemia and intra-uterine growth restriction [14–17].

Bed nets are typically distributed via antenatal and immunization clinics in order to reach high-risk populations: pregnant women and children under 5 years old. Because women often enroll in antenatal care late in pregnancy, interventions targeting all women of child-bearing age have the potential to protect against malaria early in pregnancy. In 2012, Malawi launched a universal bed net campaign. In Blantyre, the site of this study, the campaign targeted 250,000 households and distributed more than 400,000 bed nets with the goal of providing a bed net for every two individuals in a household. On a national level, in 2010 58 % of households had an ITN; in 2012 55 % of households had an ITN; and in 2014 70 % of households had an ITN. Universal ITN coverage (in which there is a bed net for every two individuals in a household) expanded from 19 to 30 % between 2012 and 2014 [18, 19]. Expansion of artemisinin combination therapy (ACT) use for treatment of malaria and rapid diagnostic testing (RDT) for malaria also increased over this period. ACT use for treatment of fever in children under five increased from 27.6 % in 2010, to 29.6 % in 2012, to 39.3 % in 2014 [18–20]. In 2010 Malawi shifted policy to include the use of RDTs for malaria diagnosis, then in 2012 there was a national roll out of RDTs to health facilities [21].

To test the hypothesis that a universal bed net campaign would reduce the burden of pregnancy-associated malaria prior to antenatal clinic attendance, data on bed net use and blood samples were collected at the first antenatal visit in clinical studies that occurred from 2009 to 2014 in Blantyre, Malawi. Malaria prevalence and reported bed net use were compared over this time period.

## Methods

### Study site and population

Data presented are from the first antenatal visit from a sequential series of studies of pregnant women conducted in Ndirande, a peri-urban township of Blantyre, Malawi. Malaria transmission occurs year-round with a seasonal peak during the 3- to 4-month rainy season. In both studies, women were screened for enrolment at their first antenatal visit if they were in their first or second pregnancy, had not received IPT and were at less than or equal to 28 weeks of gestation. Women were excluded if they had any major illness requiring high-risk obstetric care, HIV infection or chronic antibiotic treatment. The first study was an observational study of malaria in pregnancy that enrolled women from June 2009 to June 2010 [13]. The second study was a randomized, controlled clinical trial of chloroquine as chemoprophylaxis versus IPT to prevent malaria in pregnancy in Malawi that enrolled women from February 2012 to April 2014 (ClinicalTrials.gov Identifier: NCT01443130). Enrolment blood samples were collected prior to any study interventions or standard antenatal care. Data on maternal age and gravidity were available from the observational study but not the clinical trial.

### Study procedures

Medical history and bed net use were recorded and physical examination, including axillary temperature, was performed. For all subjects, regardless of symptoms, finger-prick filter papers were collected and subjected to quantitative real-time PCR (qPCR) for *Plasmodium falciparum* lactate dehydrogenase or 18 s rRNA to detect infection. Extraction and PCR protocols are described online [22]. If the subject had signs or symptoms suggestive of malaria, thick and thin blood smear was prepared and read in real-time. Malaria smears were Field or Giemsa-stained and examined using light microscopy with a 100× oil immersion lens. Parasitaemia was assessed by counting the number of parasites per 200 leukocytes and examining 100 high-power fields before considering a smear to be malaria-negative. All slides were read by two microscopists, and in cases of disagreement between the readings, the conflict was adjudicated by a third expert reader. Parasite density was calculated based on 8000 leukocytes per microlitre.

Ethical approval was obtained from the University of Malawi College of Medicine’s Research Ethics Committee and the University of Maryland Baltimore Institutional Review Board. Written informed consent was obtained from all participants before conducting any study-related activities. Participants had the option to withdraw from the study at any time. All data were recorded and analysed anonymously.

### Definitions

The rainy season was defined as 1 December to 31 March and the dry season was defined as 1 April to 30 November. Clinical malaria was defined as parasites detected by light microscopy and associated symptoms including at least one of the following: objective fever measured at the clinic, history of fever in the previous 48 h or other symptoms in the previous 48 h including headache, myalgia, vomiting, or weakness. Malaria infection was defined as parasites detected by molecular methods, regardless of symptoms. Bed net use was determined based on the participant’s report of having slept under a bed net the previous night. Women also reported the number of nights in the previous month that they slept under a bed net. Frequency of bed net use was classified into three categories: slept under a bed net every night in the last month, slept under a bed net some nights in the last month, or never slept under a bed net during the last month. Gestational age was estimated based on last menstrual period for the 2009–2010 study and based on ultrasound for the 2012–2014 study.

### Data analysis

Data analysis was performed using Stata version 12.0 software (StataCorp, College Station, TX, USA). Graphs were produced using GraphPad Prism version 5.01 for Windows (GraphPad Software, San Diego, CA, USA). Descriptive statistics were analysed using frequencies and proportions for categorical data and geometric means, means, standard deviations, and 95 % confidence intervals for continuous variables; 95 % confidence intervals were calculated for all proportions and prevalence rates. To measure the association between gravidity, maternal age, gestational age, seasonality or bed net use and malaria infection or clinical malaria, logistic regression was used to calculate odds ratios. P > 0.05 was considered statistically significant.

## Results

### Bed net use increased following a universal bed net campaign

**Fig. 1:**
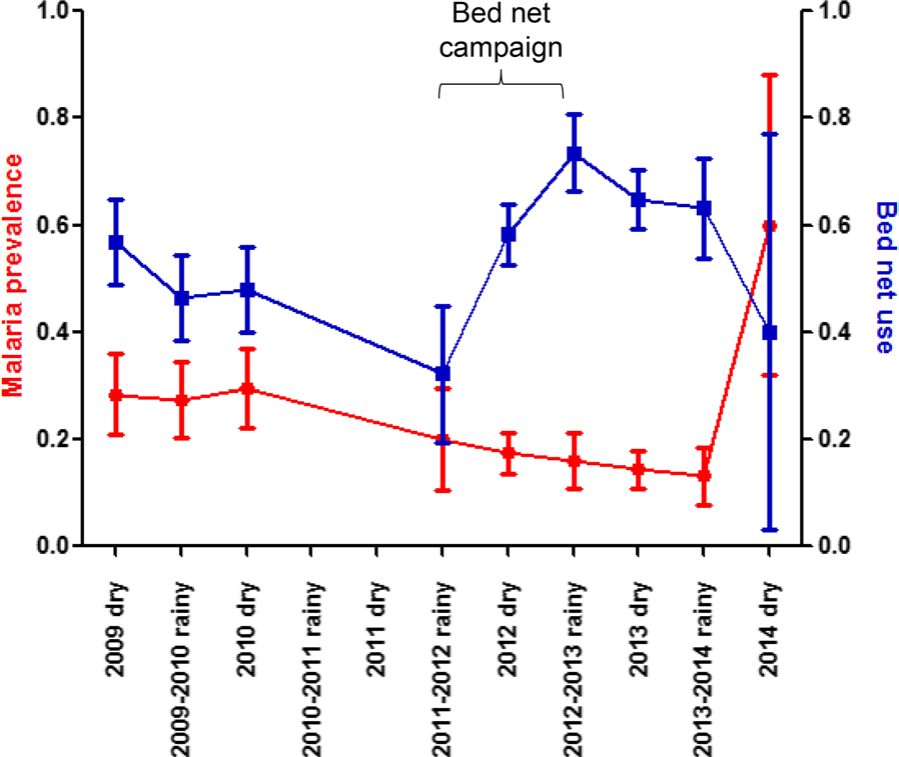
Malaria infection prevalence and bed net use by season. The proportion of individuals with malaria at the time of first antenatal visit is shown on the *left-side* Y-axis in *red*. The proportion of individuals who reported sleeping under a bed net the previous night is shown on the *right-side* Y-axis in *blue*. Data show mean and 95 % confidence interval

Some 1661 women were screened at their first antenatal visit for two studies from 2009 to 2014. Data on bed net use were available for 1347 individuals. Overall, 57.8 % (779/1347) of women reported using a bed net at their first antenatal visit. Bed net use increased from 50.3 % (225/447) before the universal campaign, to 54.8 % (200/365) the year of the campaign, to 66.2 % (354/535) following the campaign (Table 1). Bed net use was highest during the rainy season of 2012–2013, immediately following the campaign (73.4 %, 105/143) and then declined (Fig. 1). There was no association between bed net use and season (OR 1.0; 95 % CI 0.8–1.2; p = 0.78). Bed net use was significantly higher after the campaign as compared to before (OR 1.9; 95 % CI 1.5–2.5; p < 0.0001). When data were examined by frequency of bed net use after compared to before the bed net campaign, a greater proportion of individuals reported constant bed net use during the last month (44.0 vs 62.7 %, p < 0.0001), a smaller proportion reported partial bed net use during the last month (8.7 vs 4.3 %, p = 0.01) and a smaller proportion of individuals reported never using a bed net (47.3 vs 33.0 %, p < 0.0001).

**Table 1:** Malaria prevalence and bed net use before, during and after a universal bed net campaign

Time period	Bed net use	OR (95 % CI)	Malaria infection prevalence	OR (95 % CI)	Clinical malaria prevalence	OR (95 % CI)
Before bed net campaign	225/447 (50.3 %)	Referent	125/440 (28.4 %)	Referent	39/449 (8.7 %)	Referent
During bed net campaign	200/365 (54.8 %)	1.2 (0.9–1.6)	87/471 (18.5 %)	0.6 (0.4–0.8)	11/364 (3.0 %)	0.3 (0.2–0.6)
After bed net campaign	354/535 (66.2 %)	1.9 (1.5–2.5)	108/718 (15.0 %)	0.4 (0.3–0.6)	16/534 (3.0 %)	0.3 (0.2–0.6)
Total	779/1347 (57.8 %)		320/1629 (19.6 %)		66/1347 (4.9 %)

### Malaria infection prevalence decreased following a universal bed net campaign

Dried blood spots on filter papers were available for qPCR from 1629 women. Overall, the malaria prevalence at first antenatal visit was 19.6 % (320/1629). Malaria prevalence over time followed the inverse pattern of bed net use. Malaria prevalence declined significantly from 28.4 % (125/440) prior to the universal bed net campaign, to 18.5 % (87/471) the year of the campaign, to 15.0 % (108/718) following the campaign (Table 1). Malaria prevalence dropped in the years following the bed net campaign and was lowest in the rainy season of 2013–2014 (13.2 %, 20/152), but rose in the dry season of 2014 (60.0 %, 9/15) (Fig. 1). This difference was significant (p = 0.0001). There was no association between malaria infection and season of antenatal visit (OR 0.9; 95 % CI 0.7–1.2; p = 0.46) (Table 2).

**Table 2:** Factors associated with malaria at the time of first antenatal visit

	Malaria infection		Clinical malaria	
	OR (95 % CI)	p value	OR (95 % CI)	p value
Season	0.9 (0.7–1.2)	0.46	0.5 (0.3–0.9)	0.02
Maternal age^a^	1.0 (0.9–1.0)	0.36	0.9 (0.8–1.0)	0.22
Gestational age	0.96 (0.92–0.996)	0.03	1.0 (0.9–1.1)	0.62
Gravidity^a^	1.2 (0.8–1.8)	0.38	0.8 (0.4–1.7)	0.64
Bed net use	0.8 (0.6–1.1)	0.23	0.7 (0.4–1.2)	0.19
Year	0.8 (0.7–0.9)	<0.0001	0.7 (0.6–0.8)	0.001

^a^Data only available for 2009–2010

### Clinical malaria prevalence

Information on clinical malaria was available for 1347 individuals. Overall, 4.9 % (66/1347) of women had clinical malaria at their first antenatal visit. The prevalence of clinical malaria dropped significantly from 8.7 % (39/449) before the bed net campaign, to 3.0 % (11/364) the year of the bed net campaign and remained at 3.0 % (16/534) following (Table 1). Among those with clinical malaria, the geometric mean parasite density was 1640 parasites per microlitre of blood (95 % CI 1098–2449).

### Protective effect of reported bed net use

Clinical malaria had no significant association with bed net use (OR 0.71; 95 % CI 0.4–1.2; p = 0.19) when analyzing individuals. There was no significant association between malaria infection and bed net use (OR 0.8; 95 % CI 0.6–1.1; p = 0.23). There was also no significant association between gravidity, gestational age or maternal age and malaria infection or clinical malaria in univariate analysis (Table 2).

## Discussion

These data show significantly increased reported bed net use, decreased malaria infection, and decreased clinical disease prevalence at first antenatal visit following a universal bed net campaign. However, despite the universal bed net campaign, malaria prevalence at first antenatal visit remained high. Overall, one in five women was infected at the time of first antenatal visit, and even in the rainy season of 2013–2014, when infection prevalence was lowest, 13 % of women tested positive even in this urban setting. A clear inverse relationship was observed between bed net use and malaria infection. The lowest infection prevalence and highest bed net use were reported in the same year. However, bed nets did not appear to provide individual level protection against malaria. This suggests that the benefit of bed net use occurs on the population level as has been noted previously in settings where community bed net use is high [23].

There are several limitations to this study. It relied on self-reported bed net use, which may have been inaccurate. While this may result in an overestimate of bed net use, this potential bias would be consistent across years and not affect the pre- and post-intervention patterns observed. All of the women enrolled were in their first or second pregnancy, and therefore at higher risk of pregnancy-associated malaria than multigravid women. Thus, the malaria infection prevalence may not be generalizable to all pregnant women. Conversely, because women presenting to the antenatal clinic after 28 weeks gestation and women not seeking antenatal care—groups with the most limited health care access—were excluded, the study population may be biased towards women with the most access to health care and likely to have lower rates of infection.

The observation of the association between bed net use and decreased malaria prevalence does not prove causality. It is difficult to examine ecological data where interventions are applied across an entire country. However, given the documented benefits of bed net use during pregnancy [23–25], observational studies are the only ethical mechanism to examine the effect of universal bed net campaigns on pregnancy-associated malaria. While a temporal association was seen between the universal bed net campaign and malaria prevalence at first antenatal visit and reported bed net use, other potential explanations for these observations cannot be excluded. It is possible that the decline in malaria infection was due to climactic variability or other anti-malarial interventions including expansion of RDT and ACT use that were applied at the same time [18–21]. Infection rates were observed to be lowest in 2013, which was also a particularly rainy year in Blantyre [26], and it has been speculated that these unusually heavy rains in January to March may have washed away mosquito larvae, resulting in lower malaria rates overall. This could explain why the malaria infection prevalence in this study rose in the dry season of 2014. Alternatively, other anti-malaria interventions in the region could explain the decline in malaria prevalence. The Ministry of Health and Roll Back Malaria partners have continuously been scaling-up malaria control efforts nationwide. In addition to the universal bed net campaign, rapid diagnostic tests were introduced and availability of artemisinin-based combination therapy was expanded [18]. The population in Blantyre frequently travels between the city and rural villages to visit extended family, so it is possible that interventions applied in any of these areas could be responsible for changes in malaria infection prevalence at first antenatal visit in Blantyre. However, this urban population likely travels to many different rural areas, so no single other location-specific intervention is likely to have modified the patterns observed.

Although observational in nature and subject to the limitations described above, these results suggest a strong link between bed net use and prevalence of malaria in the pregnant population. There was a consistent inverse relationship between bed net use and malaria infection prevalence, including in 2014 when bed net use declined and malaria infection prevalence increased. This is consistent with intervention fatigue following a one-time campaign and the limited lifespan of bed nets. These data are mirrored by nationwide data on malaria prevalence in children under five collected as part of the Malaria Indicators survey in which microscopy-detectable malaria fell sharply from 43 % in 2010 to 28 % in 2012 and then rose slightly to 33 % in 2014 [19].

The fact that there is no individual-level association between malaria infection and bed net use is consistent with a population-level effect as has been demonstrated in other studies of bed net distribution [27, 28]. Pregnant women may have less exposure to infected *Anopheles* bites as a result of widespread availability of bed nets in the community, leading to lower transmission throughout the area.

Previous studies have clearly demonstrated the need to reach pregnant women early in their pregnancy, before malaria infection occurs. This is challenging since it is typical for women in sub-Saharan Africa to delay antenatal care until late in pregnancy [9, 10] potentially allowing infection to persist for months with adverse consequences for mother and fetus. Malaria infection in the first trimester is associated with low infant birth weight, intra-uterine growth restriction and maternal anaemia [14–17]. Additionally, modelling studies suggest that the highest rates of malaria infection and placental infection occur towards the end of the first trimester of gestation [29–31]. These infections may also constitute a significant source of malaria transmission [11]. In summary, malaria infection early in pregnancy has significant public health implications. The results of this study demonstrate that interventions that target the whole community have a significant impact on malaria prevalence in pregnancy prior to seeking out antenatal care. However, the protection is inadequate. Promotion of antenatal attendance early in pregnancy could alleviate this burden, especially if new IPT interventions could be developed that are safe in early pregnancy. Early initiation of antenatal care would also have the additional benefits of earlier access to HIV and other sexually transmitted infection testing and treatment, iron-folate supplements and detection of high-risk conditions.

## Conclusion

A universal bed net campaign was associated with a reduced prevalence of malaria during pregnancy at the first antenatal visit. These results suggest that the expansion of interventions implemented universally may be required to reduce the burden of pregnancy-associated malaria.

## Abbreviations

ACT: artemisinin combination therapy
IPTp: intermittent preventive treatment in pregnancy
ITN: insecticide-treated net
OR: odds ratio
qPCR: quantitative PCR
RDT: rapid diagnostic test
rRNA: ribosomal ribonucleic acid
